# A targeted transforming growth factor-beta (TGF-β) blocker, TTB, inhibits tumor growth and metastasis

**DOI:** 10.18632/oncotarget.24562

**Published:** 2018-02-24

**Authors:** Changhua Zhou, Jing Li, Limin Lin, Rui Shu, Bin Dong, Donglin Cao, Qing Li, Zhong Wang

**Affiliations:** ^1^ School of Pharmaceutical Sciences, Sun Yat-Sen University, Guangzhou, 510006, China; ^2^ Center for Cellular & Structural Biology, Sun Yat-Sen University, Guangzhou, 510006, China; ^3^ Ying Rui Inc., Guangzhou, Guangdong, 510009, China; ^4^ School of Biosciences and Biopharmaceutics, Guangdong Pharmaceutical University, Guangzhou, Guangdong, 510009, China; ^5^ Department of Laboratory Medicine, Guangdong Second Provincial General Hospital, Guangzhou, 510317, China

**Keywords:** TGF-β, TGF-β inhibitors, cancer metastasis, receptor, RGD

## Abstract

Transforming growth factor beta (TGF-β) promotes cancer growth in late stage cancers. To inhibit the TGF-β pathway, we investigated a tumor-targeting TGF-β receptor blocker, TTB, and its role in tumor progress. The targeted TTB comprised of the extracellular domain of the TGF-β receptor II, the endoglin domain of TGF-β receptor III, and the human immuno-globin IgG1 constant fragment (Fc). To enhance tumor microenvironment targeting, a RGD peptide was fused at the N-terminal of TTB. The targeted TTB exhibited potent TGF-β neutralization activities, and inhibited cancer cell migration and invasion as well as colony formation. In xenograft models, the TTB had potent tumor inhibition activities. The TTB also attenuated the TGF-β1-induced Smad2 phosphorylation and epithelial to mesenchymal transformation (EMT), and suppressed breast cancer metastasis. Thus, the TTB is an effective TGF-β blocker with a potential for blocking excessive TGF-β induced pathogenesis *in vivo*.

## INTRODUCTION

Transforming growth factor beta (TGF-β) is a multifunctional cytokine including three different isoforms, TGF-β1, TGF-β2 and TGF-β3. These isoforms differ in their binding affinity to three main cell surface TGF-β receptors (TGF-βR), TGF-β RI, RII and RIII, that regulate a diverse of cellular processes. TGF-β RI and RII belong to serine/threonine kinase receptors, whereas RIII, also known as beta-glycan (BG), binds and enhances the binding of TGF-β, especially TGF-β2, to RII [1]. TGF-β signals by facilitating the formation of a heterodimeric complex of TGF-β RII and TGF-β RI [2]. After TGF-β RII is activated by TGF-β binding, it binds and transphosphorylates TGF-β RI to stimulate kinase activity. The activated TGF-β RI phosphorylates transcriptional factor Smad2 or 3. The phosphorylated Smad2 or 3 then bind to Smad4, and regulate the transcription of many downstream genes [3, 4].

TGF-β regulates many diverse functions, including the proliferation and differentiation of cells, embryonic development and wound healing [3, 5]. TGF-β also regulates extracellular matrix formation and angiogenesis [6, 7], and down-regulates both primary and secondary immune responses [8–10]. In tumor development, TGF-β can have opposite roles [11]. In the early stage cancer development, TGF-β is considered as a negative regulator of cell proliferation. However, in late stage cancers, cancer cells become resistant to growth inhibition by TGF-β, and secret higher levels of TGF-β [12], which enables cancer cells become more invasive and metastasize to the surrounding organs [13]. TGF-β can also facilitate tumor progression by inhibiting immunosuppressive activities [14] and stimulates angiogenesis [5, 6, 13, 15]. Thus, inhibiting TGF-ß has been proposed to combat malignant tumors.

Considering the broad role of TGF-β in late stage tumor progression, many approaches have been taken to inhibit TGF-β activity, including TGF-β-neutralizing antibodies [16–18], small molecular inhibitors of TGF-β RI [19] and soluble TGF-β receptor trap [20]. For example, TGF-β–neutralizing antibodies and the TGF-β RI kinase-specific inhibitor (SD208) can decrease myeloma cell growth and cell adhesion to the bone marrow stromal cells [19]. GC1008, a human monoclonal antibody, which can neutralize all three human isoforms of TGF-β, inhibited tumor progression with no dose-limiting toxicity [21]. TGF-β inhibition can also block cancer metastasis. In B16 murine melanoma model, anti-TGF-β therapy decreased lung metastases [22]. The neutralizing TGF-β antibody 1D11 has also been shown able to inhibit metastasis in preclinical models of breast cancer and melanoma [23].

Previously, the fusion of TGF-β binding domains of RIII and RII has shown promising results in preclinical models by blocking TGF-β activity [1]. However, a potential issue with this fusion is its short half-life. Another issue with current TGF-β inhibitors is the systemic inhibition of TGF-β. As TGF-β plays multiple roles in different tissue and cellular contexts, toxicities are associated with the non-targeting TGF-β inhibitors. To improve tumor microenvironment targeting, RGD peptides have been proposed [24, 25]. RGD (Arg-Gly-Asp) peptides have a strong binding and high selectivity to integrins, which mediate cell-to-cell and cell-to-matrix interactions. Integrins are also frequently over-expressed on many tumor cell types and on endothelial cells of tumor-associated angiogenesis [26].

In this report, we presented a targeted TGF-β blocker (TTB) by fusing the minimal TGF-β binding domains of TGF-β receptor type II and III at the c-terminal of human IgG1 Fc. The Fc portion can enhance the half-life in patients and facilitate easy purification. The dimerization of Fc also increased the valency of TGF-β trap from monovalent to bivalent. To facilitate the tumor microenvironment targeting, a RGD peptide was fused at the N-terminal of TTB. Our data showed that TTB has strong neutralizing activity against all three TGF-β isoforms. In a diverse cellular and animal models, TTB exhibited strong anti-tumor and anti-metastatic activities, demonstrating that TTB may have a potential in human cancer therapy.

## RESULTS

### The targeted pan-TGF-β blocker TTB strongly neutralizes TGF-β isoforms *in vitro*

**Figure 1:**
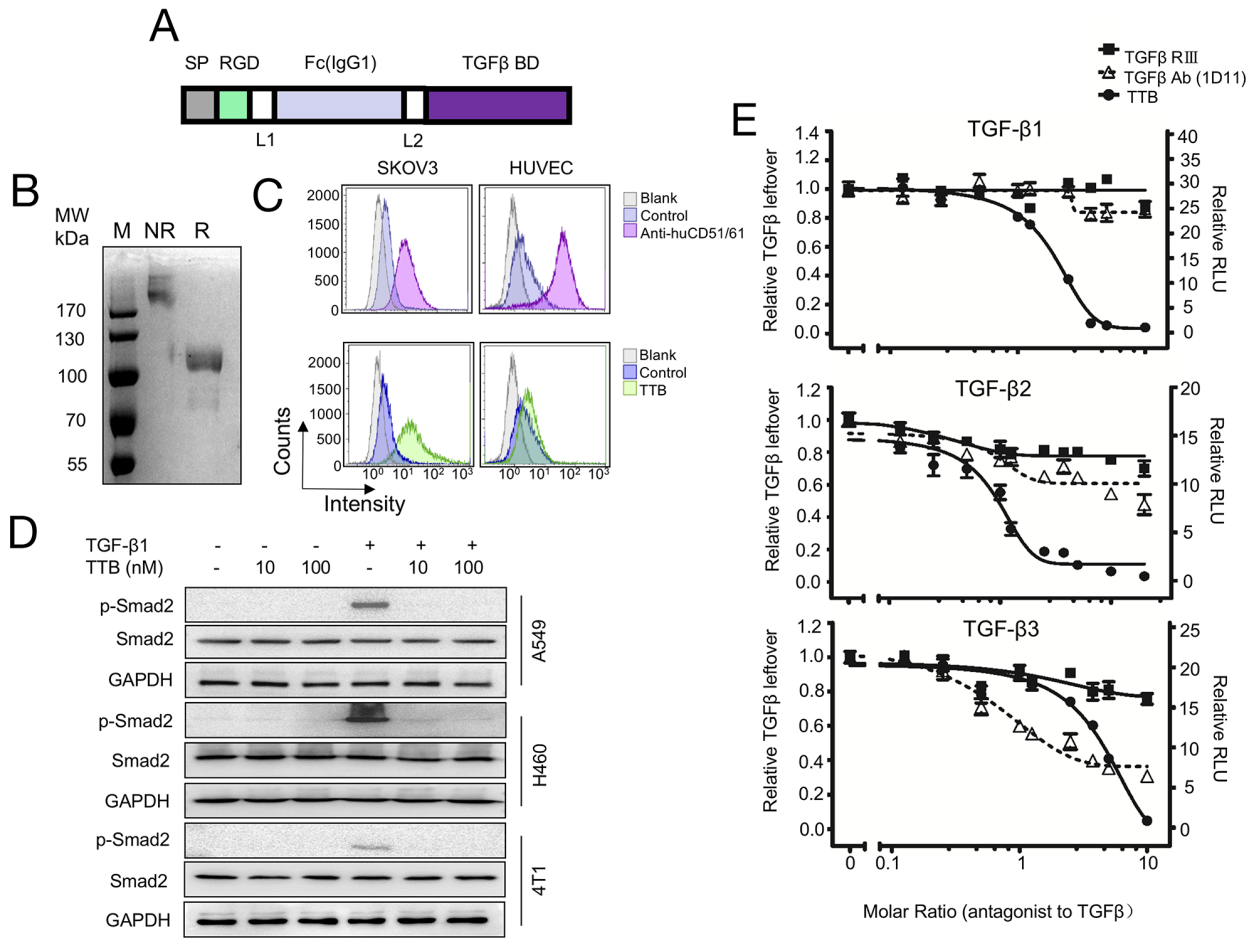
The targeted TGF-β blocker TTB strongly neutralizes TGF-β isoforms *in vitro* **(A)** Schematic representation of TTB. SP, signal peptide; Fc, CH2 and CH3 of human IgG1; TGF-β BD, TGF-β binding domain (TGFRII fragment + TGFRIII fragment); L1 and L2, GGGGS. **(B)** Coomassie blue staining of purifed TTB by SDS-PAGE under non-reducing (NR) and reducing (R) conditions. **(C)** Flow cytometry analysis of TTB binding to α_V_β_3_ integrin on SKOV3 and HUVEC cells. Anti-α_V_β_3_ integrin (CD51/61) is in the upper panel and TTB is in the lower panel. **(D)** Effect of TTB on TGF-β induced Smad2 phosphorylation. A549, H460 and 4T1 cells were treated with or without TGF-β1 (20pM), and different concentrations of TTB for 2 hrs. Western blots were then performed to detect the p-Smad2, Smad2, and GAPDH. **(E)** Neutralization of different TGF-β isoforms by TTB. The engineered MLEC cells with TGF-β luciferase reporter gene were treated with 80 pM recombinant TGF-β1, 2, or 3 in the presence of different concentrations of TTB, sTβRIII, and pan TGF-β mAb (1D11). Luciferase reporter assays were performed as described in the Methods and Materials. The data are shown as the mean ± SEM from representative of three independent experiments.

To construct the targeted pan-TGF-β blocker (TTB), the TGF-β binding domains in TGF-β receptor type II and III were fused together to block all three TGF-β isoforms [1]. To minimize the length of the construct, the minimal TGF-β binding domains in TGF-β receptor type II (aa phe73-Asp184) and III (aa Gly21-Asp379) were fused together with a GGGGS linker between (Figure 1A). To enhance the *in vivo* half-life and tumor targeting, an RGD peptide and the Fc portion of human IgG1 were fused at the N-terminal (Figure 1A). The signal peptide of IL-2 was added to the N-terminal of the construct for secreted expression. The fusion TTB was subsequently cloned into pCDNA3.1(+) vector and transiently transfected into 293F cells. TTB protein was purified from the supernatant culture medium by protein A affinity purification with yields of about 20 mg/L. Under reducing condition (R), a very weak smear band was seen at 85kDa (the predicted molecular weight), while majority of protein ran at approximately 110kDa, suggesting that most of purified TTB underwent post-translational modification such as glycosylation (Figure 1B). Under non-reducing conditions (NR), TTB was most over 170kDa, suggesting a dimeric form (Figure 1B). Gel filtration analysis also confirmed similar results (Supplementary Figure 1A). Furthermore, TTB also displayed minimal radius change even at temperature up to 85 °C, suggesting good thermo-stability (Supplementary Figure 1B).

To check whether TTB can bind the tumor microenvironment associated integrins, flow cytometry assay was performed to analyze whether TTB can bind to tumor cells SKOV3 and human umbilical vein endothelial cells, HUVEC, which have high expression of α_V_β_3_ integrins [24, 27]. TTB can indeed bind SKOV3 cells and HUVEC (Figure 1C), suggesting the RGD peptide is functional.

To analyze whether TTB can neutralize TGF-β activity, TGF-β induced Smad2 phosphorylation was checked [28]. When cancer cell lines A549, H460, or 4T1 were treated with TGF-β1, increased Smad2 phosphorylation (pSmad2) was observed (Figure 1D). When the cells were then treated with TTB, Smad2 phosphorylation was completely abolished (Figure 1D), suggesting that TTB can block TGF-β induced Smad2 phosphorylation.

Luciferase reporter assay was then performed using the modified MLEC cells to further analyze the neutralization activity of TTB [1]. When the MLEC reporter cells were treated with TGF-β1, TGF-β2, or TGF-β3, luciferase activities were induced (Figure 1E). When the cells were then treated with TTB, decreased luciferase activities were observed in all three TGF-β isoform treatments (Figure 1E). As controls, the commercially available sTGF-βRIII and TGF-β mAb (1D11) were also analyzed in this assay. TTB displayed better neutralization activity for all 3 different TGF-β isomers (Figure 1E), with IC_50_ values of 144.08 pM, 67.92 pM and 313.92 pM in the presence of 80 pM for TGF-β1, TGF-β2 and TGF-β3 respectively.

### TTB suppresses colony formation *in vitro*

**Figure 2:**
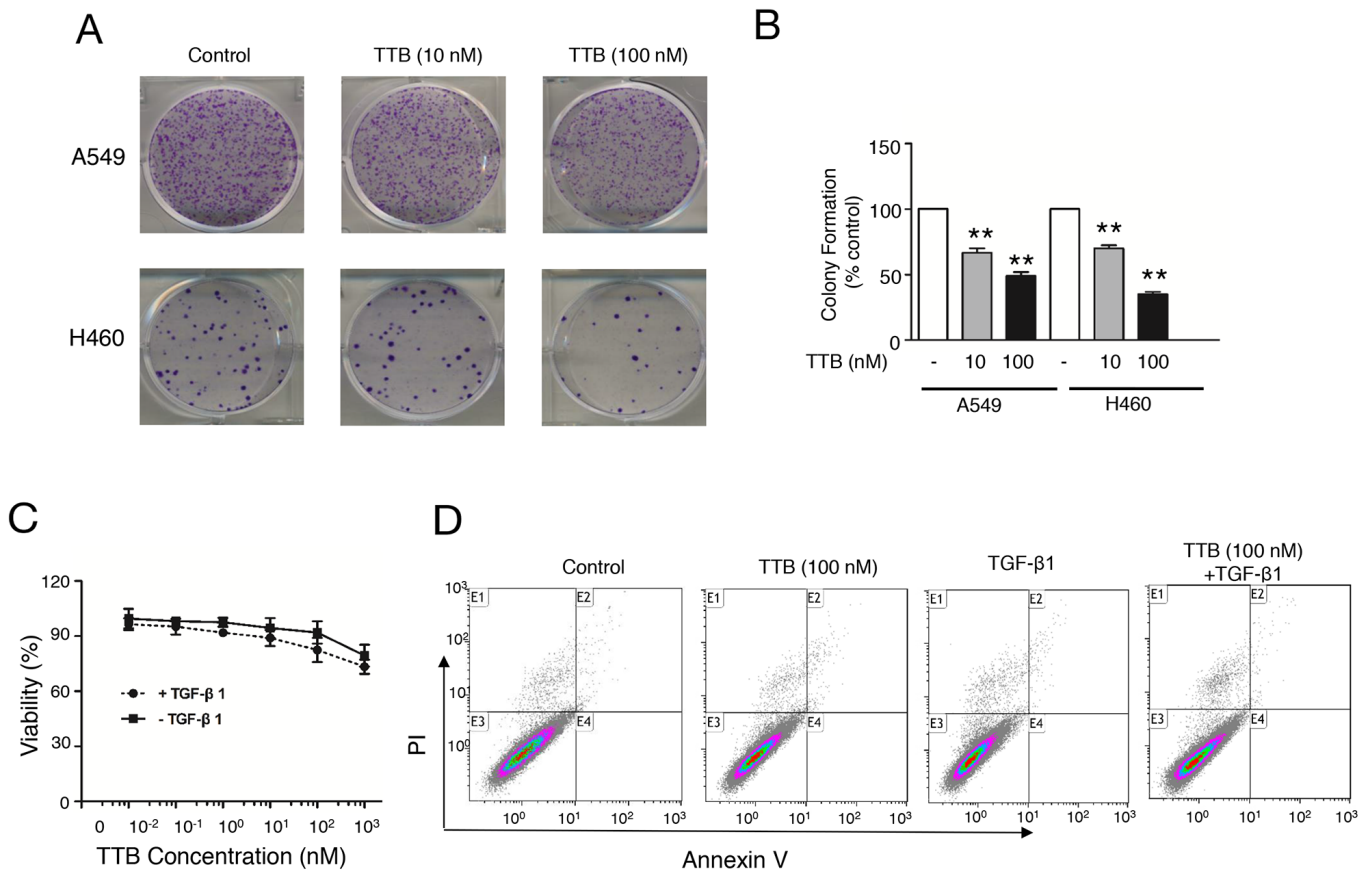
TTB suppresses colony formation *in vitro* **(A)** Colony formation assay using A549 and H460 were performed as described in the Methods and Materials. **(B)** Bar graph of colony formation assay results by counting the number of colonies containing over 50 cells. The bars represent the normalized values to control groups. The data are shown as the mean ± SEM with ^**^P<0.01 vs. TTB (0 nM) group. **(C)** Cell viability, and **(D)** Cell apoptosis were evaluated for A549 cells cultured in petri dish with indicated concentrations of TTB with or without TGF-β1 (20 pM).

To study the effect of TTB on cancer cells, colony formation assays were performed using lung cancer cell line A549 and H460 cells. For both H460 cells and A549 cells, TTB can significantly decrease colony formation (Figure 2). In A549 cells, TTB reduced the colony formation to an average of 66.63% in 10 nM treatment group and 48.94% in 100 nM group (Figure 2A, 2B). The same inhibition effects can also be found in H460 cells, with an average of 70% in 10 nM group and 35% in 100 nM group (Figure 2A, 2B). These data suggested that TTB can suppress cancer cell colony formation *in vitro*.

To further investigate whether TTB has any effect on tumor cell growth, A549 growth on petri dish was also tested *in vitro*. TTB treatment in the presence or absence of 20 pM TGF-β1 has no effect on A549 cell proliferation (Figure 2C). Similar results were also observed when the cells were cultured in serum free medium (Supplementary Figure 2A). Furthermore, no apoptosis was observed by TTB with or without TGF-β1 treatment by flow cytometry analysis using Annexin V-FITC and propidium iodide (PI) (Figure 2D). TTB or TGF-β1 did not affect cell cycle either (Supplementary Figure 2B). TTB had no effect on cell growth and apoptosis in H460 cells either (data not shown). Taken together, these results demonstrated that TTB had no effect on cell cycle in the current culture conditions.

### TTB inhibits cancer cell migration

**Figure 3:**
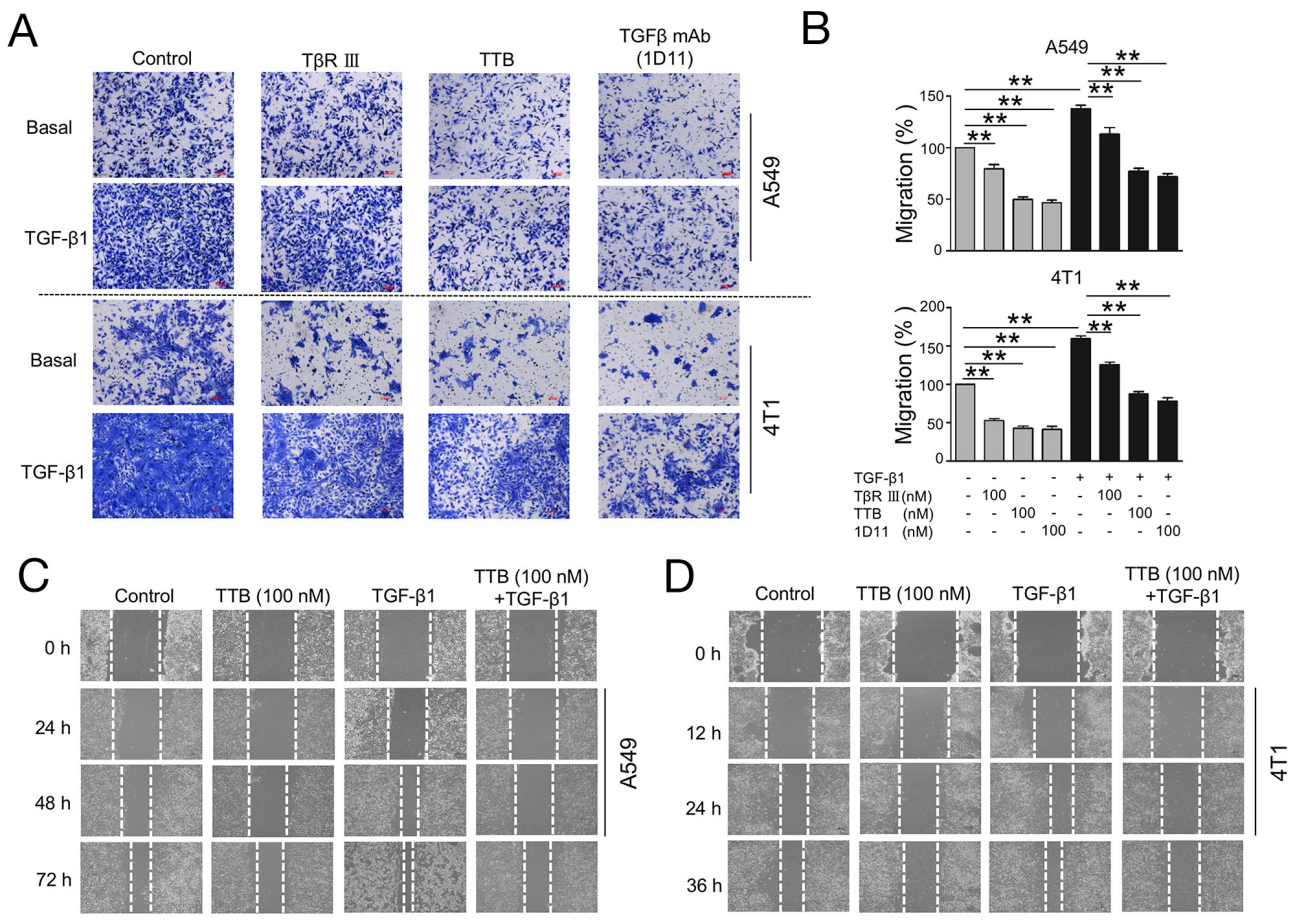
TTB inhibits cancer cells migration **(A)** Migration assay was performed as described in the Methods and Materials. A549 and 4T1 cells were cultured alone, ,100nM of TβRIII, TTB or TGFβ mAb(1D11), with or without TGF-β1 (20pM). Images are representative of at least 3 independent experiments. **(B)** Bar graph of the migration assay. The data represented the normalized values of the number of migrated cells to control groups. The data are shown as the mean ± SEM with ^**^P<0.01. **(C)** and **(D)** Scratch-wound assay of A549 and 4T1 cells.

As TGF-β plays an important role in the cell migration, especially metastatic tumor progression [28], the function of TTB in regulating cellular migration was explored using transwell assays. Lung cancer cell A549 can migrate efficiently in the transwell assay (Figure 3A, 3B). Exogenous TGF-β1(20 pM) induced more robust cell migration (Figure 3A, 3B). The addition of TTB (100nM) inhibited the migration of the A549 cells with or without the exogenous TGF-β1 (Figure 3A, 3B). TTB was equivalent to TGF-β mAb (1D11), probably slightly better than sTGF-βRIII in inhibiting migration. The similar inhibition can also be found in 4T1 cells (Figure 3A, 3B). These data suggested that TTB can reduce tumor cell motility *in vitro*.

To further examine the role of TTB in inhibiting cell migration, the scratch-wound healing assay was performed. A549 and 4T1 cancer cells were used in this assay. Over a 72h period, TGF-β1 promotes the migration (Figure 3C). A549 cells treated with TTB migrated significantly slower than TGF-β1 treatment or control (Figure 3C). After 72 h, A549 cells treated with 100 nM TTB were 57.14 % closed, while untreated control were 42.86%. A549 cells treated with the combination of 100nM TTB and TGF-β1 were 52.38 % closed, whereas TGF-β1 treatment alone was 18.18%. The similar inhibition activities can also be found in 4T1 cells (Figure 3D).

The effect of TTB on cancer cell migration was also analyzed using xCELLigence DP Real-Time Cell Analyzer (RTCA). For both human lung cancer cell line A549 and murine breast cancer cell line 4T1, TTB can inhibit the cell migration in this assay (Supplementary Figure 3A, 3B). Collectively, these data showed that TTB can inhibit cell migration *in vitro*.

### TTB inhibits cancer cell invasion and attenuates TGF-β1-induced EMT

**Figure 4:**
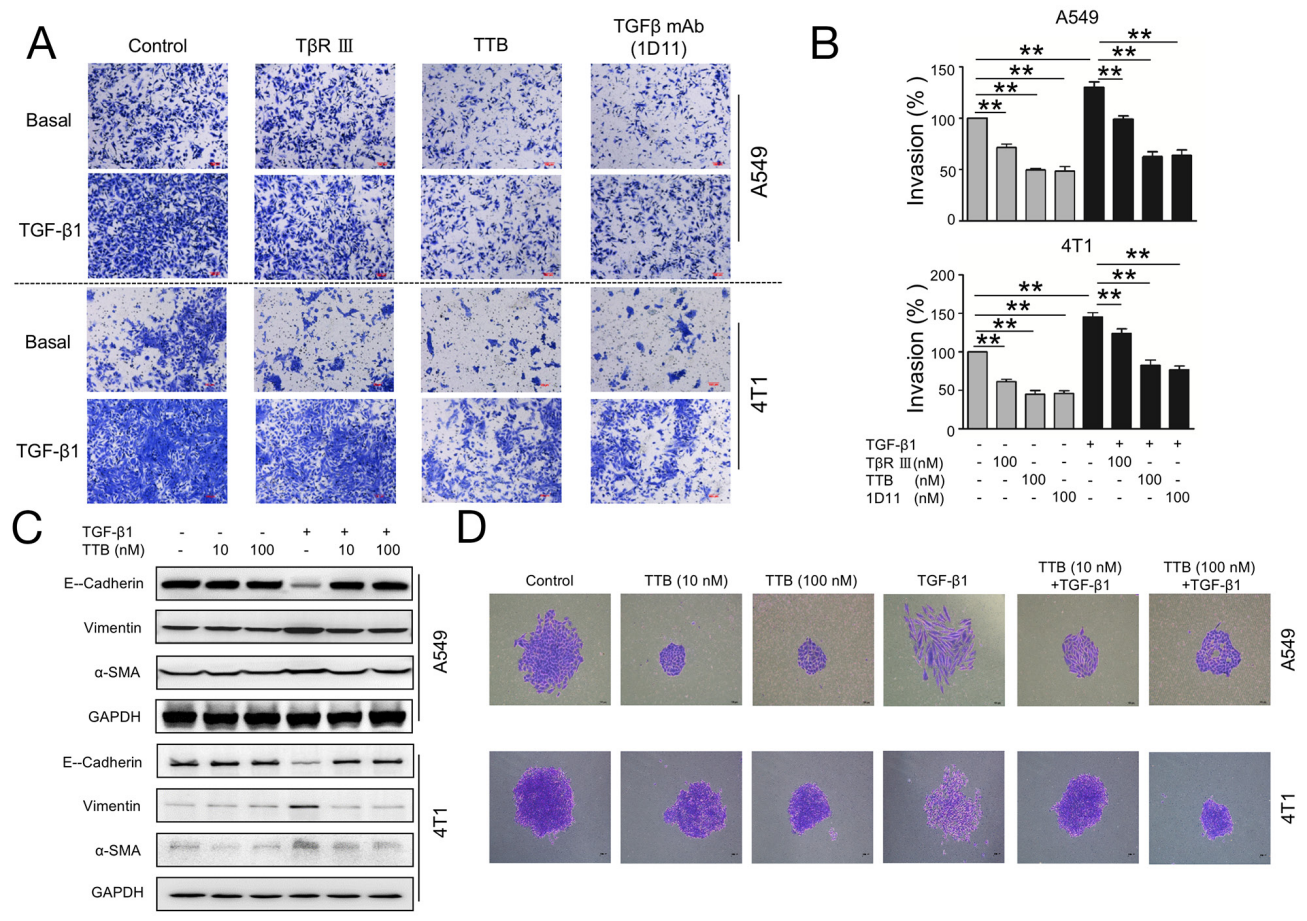
TTB inhibits cancer cells invasion and EMT **(A)** Invasion assay was performed as described in the Methods and Materials. A549 and 4T1 cells were cultured alone, 100nM of TβRIII, TTB or TGFβ mAb(1D11), with or without TGF-β1 (20pM).. Representative images were shown. **(B)** Bar graph of the invasion assay. The data represented the normalized values of the number of invaded colonies against control groups. The data are shown as the mean ± SEM with ^**^P<0.01. c). **(C)** The biomarker expression during TGF-β1 treatment. A549 and 4T1 cancer cells were incubated with TGF-β1 in the presence or absence of TTB for 2 days. The protein levels of E-Cadherin, Vimentin and α-SMA, were determined by Western blot. **(D)** Single A549 or 4T1 cancer cell was incubated with TGF-β1 in the presence or absence of TTB for 7 days and 2 days, respectively. The cells were then fixed and images obtained under a microscopy at 100 × magnification. Representative images were shown here.

One essential element for cancer metastasis is the invasion through basement membrane. To study the cancer cell invasion *in vitro*, cellular matrix Matrigel was used to mimic the basement membrane. TTB significantly inhibited A549 invasion by 50.50 % comparing with the control group (Figure 4A, 4B). TGF-β1 increased invasion of A549 cells by an average of 30.35% and such increase can be neutralized by TTB (Figure 4A, 4B), similar to TGF-β mAb (1D11), and better than sTGF-βRIII. The similar results can also be found in 4T1 cells (Figure 4A, 4B). The effect of TTB on cancer cell invasion was also analyzed with coated Matrigel using xCELLigence DP Real-Time Cell Analyzer (RTCA). For both A549 and 4T1 cells, TTB can suppress the cell invasion (Supplementary Figure 3C, 3D). Taken together, these results suggest that TTB decreases both basal and TGF-β1-stimulated invasion.

A critical contributor to the metastatic process is epithelial to mesenchymal transition (EMT), which is associated with the loss of epithelial marker, such as E-Cadherin, and gain of mesenchymal markers, including Vimentin and α-SMA [29]. The human lung cancer cell line A549 has been reported to undergo EMT in response to TGF-β1 [30]. The function of TTB during TGF-β1-induced EMT was then investigated using A549 cells. When A549 cells were treated with TGF-β1 for 48 h, reduced expression of E-cadherin, and increased expression of Vimentin and α-SMA, were observed (Figure 4C). When the cells were further treated with TTB, these changes were completely reversed (Figure 4C). Along with the biomarker expression changes, cellular morphologies were also changed. When A549 cells were treated with TGF-β1 treatment for 7 days, the cellular morphology changed from cuboidal shape to elongated spindle-like (Figure 4D). Consistent with changes of the biomarker expression, TTB reversed the morphology changes induced by TGF-β1 (Figure 4D), supporting that TTB inhibited the TGF-β1-induced EMT. Similar blockage activities were also observed in 4T1 cells (Figure 4C, 4D).

### TTB inhibits tumor growth and metastasis

**Figure 5:**
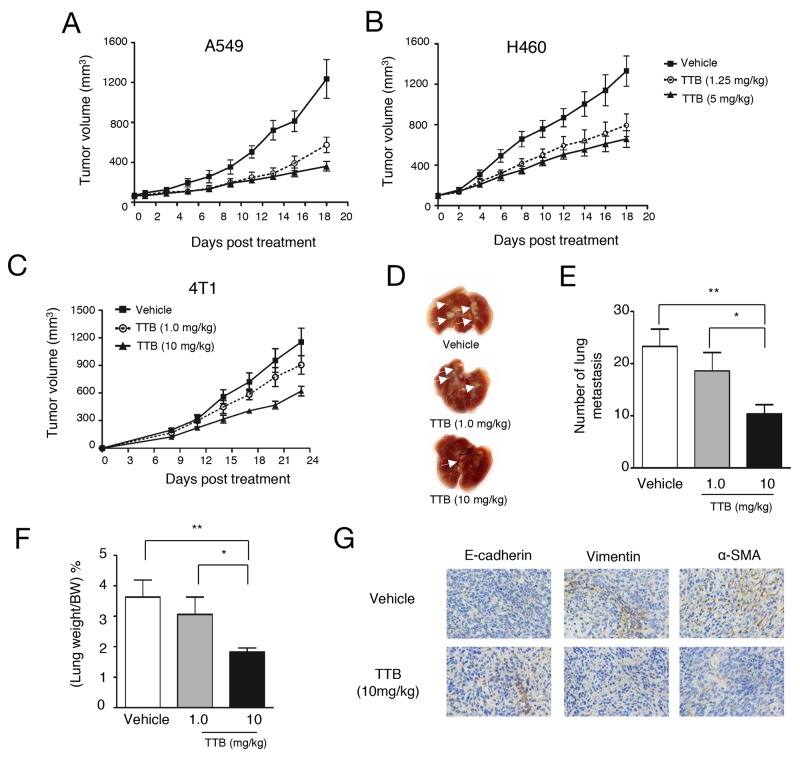
TTB inhibits tumor growth and metastasis Tumor growth inhibition studies were performed as described in the Methods and Materials. **(A)** A549 and **(B)** H460 cells were injected to into athymic nude mice (n = 8). When tumor volume reached ∼100 mm^3^, mice were intraperitoneally (i.p.) treated with vehicle or TTB every 3 days. The tumor volumes are shown as the mean ± SEM (Vehicle, solid line with square; TTB 1.25mg/kg, dot line with circle; TTB 5mg/kg, solid line with filled circle). For tumor metastasis studies, 4T1 cells were transplanted into the right-side fourth mammary gland of female BALB/c mice (n = 8). Starting from day 1, the mice were i.p. treated with vehicle or TTB every 3 days. **(C)** Growth of the tumors was measured and expressed as (length × width× width) × 0.5. **(D)** The representative images of the lungs. The lungs were fixed and white arrows indicated the lung metastatic lesions. **(E)** The lung metastasis number and **(F)** the percentage of lung weight/bodyweight were determined. **(G)** IHC staining of E-cadherin, vimentin and α-SMA expression in 4T1 primary tumor specimens. Experiments have been repeated at least 3 times. The data are shown as the mean ± SEM. ^*^P<0.05, ^**^P<0.01.

TGF-β inhibitors have been shown previously able to inhibit tumor growth *in vivo* [31]. To analyze the anti-tumor activity of TTB, A549 and H460 xenograft models in nude mice were studied. In both tumor xenograft models, TTB treatment given every 3 days can significantly inhibit tumor growth compared with vehicle group (Figure 5A, 5B), However, no obvious difference among two different dosages (1.25mg/kg and 5mg/kg), suggesting the pharmacodynamics of TTB needs to be explored in more details.

As TGF-β not only affects tumor cell behavior, but also regulate multiple functions of immune system [32], immune-competent BALB/c mice bearing CT26 tumor cells was also used to evaluate the effects of TTB. In this model, significant tumor growth inhibition (58%, p<0.05) was observed when mice were treated with 5 mg/kg TTB, but no tumor growth inhibition at the lower dosage of 1.25mg/kg (Supplementary Figure 4A). Tumor growth inhibition was also observed on C57BL6 mice bearing B16-F10 (Supplementary Figure 4B). These results confirmed that TTB has tumor growth inhibition activities.

TGF-β plays important role in late stage tumors including promoting tumor metastasis. The *in vitro* results above suggested TTB can inhibit several aspects of tumor metastasis, including cell migration, invasion and EMT. The effects of TTB on tumor metastasis was then studied using 4T1 metastatic breast cancer model. After orthotopically transplanted into the mammary fat pad, mouse 4T1 cells gradually disseminate to distant organs, predominantly to the lungs. With TTB treatment, tumor volumes in the fat pad were decreased (Figure 5C). Moreover, the number of nodules, indication of lung metastasis, were decreased in a dosage dependent manner (Figure 5D, 5E), as well as lung weight (Figure 5F).

To further investigate the hypothesis that TTB prevents cancer metastasis, immune-deficient nude mice were injected with 4T1 breast cancer cells directly into the left cardiac ventricle. The mice were then treated with TTB and sacrificed on day 10. Compared to normal lungs, 4T1 transplanted mice have increased number of lung nodules and weight ((Supplementary Figure 5A, 5B, 5C). With TTB treatment, the number of lung nodules ((Supplementary Figure 5A, 5B) and lung weights (Supplementary Figure 5C) were reduced significantly. Furthermore, TGF-β level in serum was induced in the 4T1 transplanted mice, but normalized by TTB treatment ((Supplementary Figure 5D). These data confirmed that TTB can inhibit tumor metastasis.

## DISCUSSION

In this report, we presented the anti-tumor effects of a targeted pan-TGF-β blocker, TTB, which is a Fc fusion with TGF-β binding domains of TGF-βRII and RIII. In a diverse cellular and animal models, TTB exhibited potent TGF-β inhibition, anti-tumor and anti-metastasis activities, supporting TTB as a promising molecule in cancer therapy.

TGFβ signaling pathway plays pivotal roles in tumor progression. TGFβ can suppress cancer cell growth and induce cancer cell apoptosis in early stage tumor development, whereas promotes tumor cell growth, survival, invasiveness, and metastasis in late stages [33]. Higher TGF-β levels in the serum and tissues are correlated with worse prognosis and decreased survival in gastric cancers [34] , colorectal cancers [35] and breast cancers [36]. Elevated TGF-β levels in the tumor microenvironment can also suppress anti-tumor immune activities [10]. Thus a variety of anti-TGF-β strategies have been developed and investigated [31], including: i). Reducing TGF-β expression, such as by administration of antisense molecules; ii). Reducing ligand–receptor interaction, such as TGF-β receptor antibodies or ligand traps (monoclonal TGF-β neutralizing antibodies, and soluble TGF-βR); and iii). Signal transduction inhibition, such as small molecule inhibitors inhibiting TGF-β, or peptide aptamers inhibiting Smad activity [31, 37].

In this study, the soluble TGF-β receptor was used to trap TGF-βs and prevent their interaction with the cell surface TGF-β receptors. Several previous studies have employed soluble TGF-β receptors to trap TGF-β and inhibit its activity, and have shown anti-tumor activities *in vivo* [20, 31, 37-42]. For example, sTβRII and sTβRIII were shown to be capable of abrogating the TGF-β activities and enhancing glioma cell lysis by NK cells [43]. Administration of sTβRII can inhibit ovarian tumor growth, and completely abolish ascites formation through inhibiting VEGF expression and normalization of lymphatic vessels [44]. sTβRII can also inhibit mesotheliomas tumor growth by enhancing the anti-tumor immune activities [45], and reduce breast cancer and pancreatic cancer metastasis [46–48]. Recently, TβRII-Fc delivered by an adenovirus (Ad.sTβRII-Fc) resulted in the blockade of the TGF-β-induced phosphorylation of Smad2 and Smad3, as well as the reduction of bone metastasis and osteolysis [49–52]. sTβRIII can also suppress the tumor formation and reduce lung metastasis via by binding TGF-β and inhibition of angiogenesis [53, 54], and inhibit prostate tumor proliferation and angiogenesis through decreasing MMP-9 expression [55]. Thus, sequestering excess TGF-β by the soluble TGF-β receptors had encouraging anti-cancer results in pre-clinical models.

Some specific cancers are known to overexpress TGF-β2, such as malignant gliomas and prostate cancer cells [56, 57]. Other types cancer overexpress all three TGF-β isoforms, for example, breast and gastric cancers [58]. Therefore, it is also essential to control all three isoforms, especially in diseases that all isoforms are involved. For example, all three TGF-β isoforms were involved in renal fibrogenesis; and the inhibition of all three TGF-β isoforms exhibited the best therapeutic effect [59].

To block all three isoforms of TGF-β, soluble TβRII-TβRIII fusion has been studied before with good *in vitro* efficacy [1]. However, the soluble TGF-β receptors studied previously still have three major caveats: 1). Short half-life. Most of these soluble TGF-β receptors are only the extracellular or TGF-β binding domains of TGF-β receptors, which usually exhibit short half-life *in vivo*. 2). Expression and purification are not easy to scale up. Most of these studies used His-tag for purification from expression in E.coli or insect cells, which increases the difficulties in scale-up manufacture. 3) Systemic targeting. As TGF-β plays opposite roles in the early and late stage cancer development, non-tumor targeting TGF-β inhibitors have more adverse effects in non-tumor tissues.

In this report, we constructed TTB, a targeted TGF-β trap, to solve the above issues. To block all three isoforms of TGF-β, TGF-β binding domains from both TβRII and TβRIII were fused together (Figure 1). Fc fusion proteins are frequently purified using Protein A affinity chromatography, making scale-up purification much easier. Furthermore, due to the dimerization of Fc, the TTB will have tetravalent TGF-β blocking activity.

To increase the tumor targeting, an RGD peptide was fused at the N-terminal of TTB. The RGD (Arg-Gly-Asp) peptides selectively bind integrins, which are over-expressed on many types of tumors and tumor microenvironment [26, 27]. TTB demonstrated specific binding to the integrin positive SKOV-3 and HUVEC cells, while not binding to the integrin negative cell line LS174T (data not shown), suggesting that the RGD peptide will likely enhance tumor targeting. However, we have not been able to observe enhanced tumor localization of TTB in the xenograft models using immunohistochemistry method, likely due to the low concentration of TTB used in the xenograft experiments. Increasing the dosage or more sensitive methods are needed to detect TTB *in vivo*. Furthermore, the effects of RGD peptide on tumor inhibition and safety *in vivo* have not been fully evaluated. Further studies with more relevant control constructs will be critical to analyze the potential advantages of TTB in clinic.

In conclusion, the combinatorial fusion protein TTB exhibited potent TGF-β inhibition, anti-tumor and anti-metastasis activities. This fusion protein overcomes the limiting issues of previous molecules by increasing the half-life and tumor targeting, presenting TTB as a powerful immunotherapeutic agent for malignant tumors.

## MATERIALS AND METHODS

### Cell culture

MLEC cells with TGF-β luciferase reporter were generously gift from Dr. Lu-Zhe Sun at University of Texas. Human cell lines, 293F, A549, and H460 cells, and mouse cell lines, 4T1, B16-F10, and CT26 cells were purchased from the Shanghai Cell Bank, Shanghai, China. All cell lines were maintained in DMEM (Gibco) supplemented with 10% fetal bovine serum (FBS, Hyclone laboratories) and 1% penicillin-streptomycin (Gibco) at 37 °C, 5% CO_2_ in a humidified incubator.

### Generation of TTB, gel filtration and dynamic light scattering analysis

To construct the targeted TGF-β blocker (TTB), the minimal TGF-β binding domains in TGF-β receptor type II (aa phe73-Asp184) and III (aa Gly21-Asp379) were fused together with the RGD peptide and Fc portion of human IgG1 at the N-terminal [27] (Figure 1A). The fusion gene TTB was synthesized (Genscript, Nanjing, China) and then cloned into pCDNA3.1(+) vector. The construct was then transiently transfected into 293F cells. TTB protein was purified from the supernatant culture medium by Protein-A-agarose affinity chromatography (GE Healthcare).

Gel filtration analysis of TTB was performed using SephacrylS-200 High Resolution (GE Healthcare) with the standard protein markers (Sigma, MWGF1000). Dynamic light scattering (DLS, DynaPro plate reader II) was used to monitor the changes in the dimensions of the TTB protein during denaturation with the temperature range from 25°C to 85 °C.

### Luciferase reporter assay

The TGF-β luciferase report assay was performed as described previously [1]. Briefly, MLEC cells were seeded to a 96-well plate (2×10^3^ cells per well). After 12 hours, the cells were treated with 80 pM recombinant TGF-β1, TGF-β2 or TGF-β3 (R&D Systems) with different concentrations of TTB, sTGF-βRIII or pan TGF-β mAb (R&D Systems, 1D11). After 18 hours’ incubation, luciferase activity was then measured using an automated reader (MD Flex station 3) and calculated as the fold induction over no TGF-β stimulation.

### Smad2 phosphorylation

To analyze the TGF-β induced Smad2 phosphorylation, cancer cell lines A549, H460 or 4T1 were grown to 80% confluent. The cells were then serum-starved for 2 h, followed by treatment with TTB only or in combination with TGF-β1 treatment for 2 h. Cells were then lysed in RIPA lysis buffer (Beyotime) and subjected to SDS–PAGE. Western blotting was performed to analyze the level of Smad2 and phospho-Smad2 (ab53100, Abcam).

### Cell proliferation assay and flow cytometry analysis

Cell proliferation was performed as described previously [60]. Briefly, A549 cells were plated in 96-well plates at 5×10^3^ cells/well. 12 hours later, the culture medium was replaced with different concentrations of TTB in the presence or absence TGF-β1 (20 pM). After incubation for 48 hours, CCK-8 assay (Dojindo) was performed to evaluate cell viability.

To analyze the apoptosis and cell cycle, A549 cells were seeded in 12-well plates at a density of 1×10^5^ cells per well. After TTB treatment with or without TGF-β1 (20 pM) for 48 h in serum free medium, cells were collected and washed twice with PBS. Apoptosis assay was performed according to the protocol of the Annexin V-FITC Apoptosis Detection Kit (BD). Cells were analyzed by flow cytometry (Beckman FC500) and assessed using Kaluza software (Beckman Coulter).

### Colony formation assay

Colony formation assay was performed as described previously [61]. Briefly, A549 cells (1,500 cells per well), H460 cells (500 cells per well) were plated in 6-well plates containing DMEM supplemented with 10% FBS. The cells were treated with TTB for 7 to 14 days. Cells were examined with a 0.02% crystal violet solution and colonies that containing over 50 cells were counted.

### Wound-healing assay

Wound-healing assay was performed as described previously with modifications [62]. Briefly A549 or 4T1 cells were plated in a six-well plate and scratched by a 200 μl tip to cause a wound. Images were then taken using a Nikon ECLIPSE Ti microscope under 100 × magnification at indicated times. Cells were maintained in 0.2% FBS media at 37°C in 5% CO_2_. The wound was calculated using Adobe Photoshop, with the 0 h time being set to 100%.

### Matrigel migration and invasion assay

The xCELLigence DP Real-Time Cell Analyzer (RTCA) equipped with a CIM-plate 16 (Roche) was used to monitor cell proliferation, migration and invasion. For the invasion experiments, the membrane was coated with Matrigel (BD Biosciences). For the migration assays, the membrane was left uncoated. Cells (5×10^3^) were starved in a serum-free media for 8 h. 10% FBS in lower chamber was used as a chemoattractant. Migration and invasion was monitored every 30 min for 48 hours with different concentrations of TTB treatment.

Invasion and migration assays were also performed as described previously [28]. Briefly, on 24-well plates, A549 or 4T1 cells (2×10^4^) were placed in 0.2 ml serum-free media onto the upper chamber of regular or Matrigel-coated transwell filters. The lower chamber was filled with 0.6 ml DMEM supplemented with 10% FBS. After 18-36 h of incubation with sTGF-βRIII or pan TGF-β mAb (1D11) or TTB in the presence or absence of 20 pM TGF-β1, the cells on the upper surface of the filter were removed, and cells underneath of the filter were fixed and stained with a 0.02% crystal violet solution. The number of cells were counted in three fields using a Nikon ECLIPSE Ti microscope at 100 × magnification.

### Induction of EMT

Induction of EMT was performed as described previously [63]. Briefly, A549 or 4T1 cells were plated at 200,000 cells per well in a six-well dish. The cells were treated with the indicated concentrations of TTB with or without TGF-β1 for 48 hours. The images of cell morphology were taken using a microscope at 100 × magnification. The cells were then lysed in RIPA lysis buffer (Beyotime). Western blot was performed using antibodies against GAPDH, E-Cadherin, Vimentin, and α-SMA (Cell Signaling Technology).

### *In vivo* efficacy studies

To analyze the anti-tumor effect of TTB using xenograft models, 4∼6-week-old female nude mice or BALB/c mice were subcutaneously (s.c.) inoculated with cancer cells at the right flanks. When tumor volume reached ∼100 mm^3^, mice were intraperitoneally (i.p.) treated with vehicle, different concentrations of TTB every 3 days. Tumors were measured in two dimensions with an electro caliber. Tumor volumes were calculated using the formula: tumor volume = length × width × width/ 2, where length represents the larger dimension and width the smaller dimension.

To study the effect of TTB on lung metastasis, the 4T1 metastatic mouse breast cancer model was used. 4T1 cells (1×10^5^ cells/mouse) in 0.1 ml PBS were inoculated into the left cardiac ventricle of female athymic nude mice. One day after the implantation, TTB or vehicle was administered by i.p. injection every 3 days. On day 10, mice were sacrificed. The lungs were isolated and the number of metastasis was counted under a light microscope (Leica M 125).

In another metastasis model, 4T1 cells were implanted (5×10^4^ cells/mouse) into the right-side fourth mammary gland of female BALB/c mice. Starting from day 1 after the implantation, the mice were treated with vehicle (PBS) or TTB (i.p., every 3 days for 23 Days). From day 8 post treatment, growth of the tumors was measured and expressed as (length × width× width) × 0.5. On day 23, mice were euthanized. The lungs of the mice were removed and metastatic tumors was determined visually. Primary tumors were fixed and histological sections were prepared. Immunohistochemistry (IHC) analysis was undertaken to monitor the expression of E-Cadherin, Vimentin, and α-SMA (Cell Signaling Technology). The images were captured on a light microscope (Leica M 125) at 400 × magnification.

### Study approval

All animal experiments were performed according to the Sun Yat-Sen University Institutional guidelines for the Care and Use of Laboratory Animals. All protocols were approved by the Sun Yat-Sen University Institutional Animal Care and Use Committee. All mice were purchased from the Animal Experiment Facility of Sun Yat-sen University.

## SUPPLEMENTARY MATERIALS FIGURES


