# Supplementary material for: A targeted transforming growth factor-beta (TGF-β) blocker, TTB, inhibits tumor growth and metastasis
Source: Oncotarget. 2018 Feb 24;9(33):23102–13. doi: 10.18632/oncotarget.24562 (PMC5955403; doi:10.18632/oncotarget.24562)
Supplement: Supplementary file 1 [file oncotarget-09-23102-s001.pdf]

A targeted transforming growth factor-beta (TGF-β) blocker, TTB, inhibits tumor growth and metastasis

SUPPLEMENTARY MATERIALS

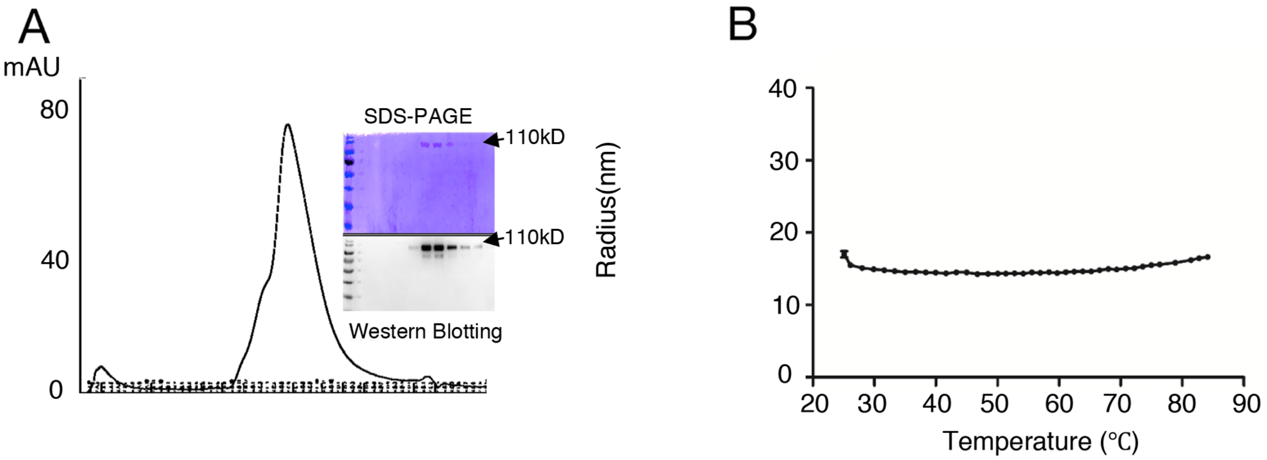

Supplementary Figure 1: Analysis of TTB protein. **(A)** Gel filtration analysis was performed to monitor the molecular weight. **(B)** Effects of the temperature on the protein particle sizes. Dynamic light scattering was performed to detect the thermo-stability.

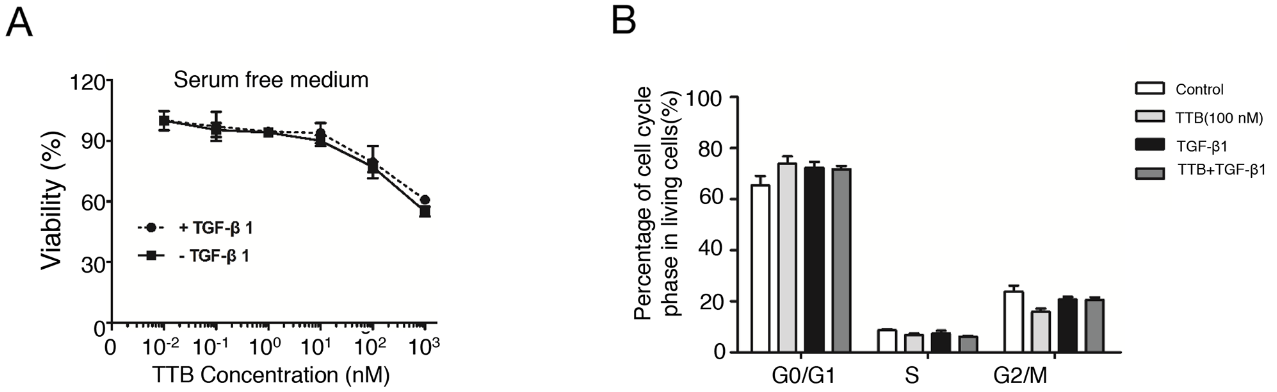

Supplementary Figure 2: **(A)** Cell viability using medium without serum, and **(B)** Cell cycle of A549 cultured in petri dish with indicated concentrations of TTB with or without TGF-β1 (20 pM).. The bars represent the normalized values to control groups. Experiments have been repeated at least 3 times. The data are shown as the mean ± SEM.

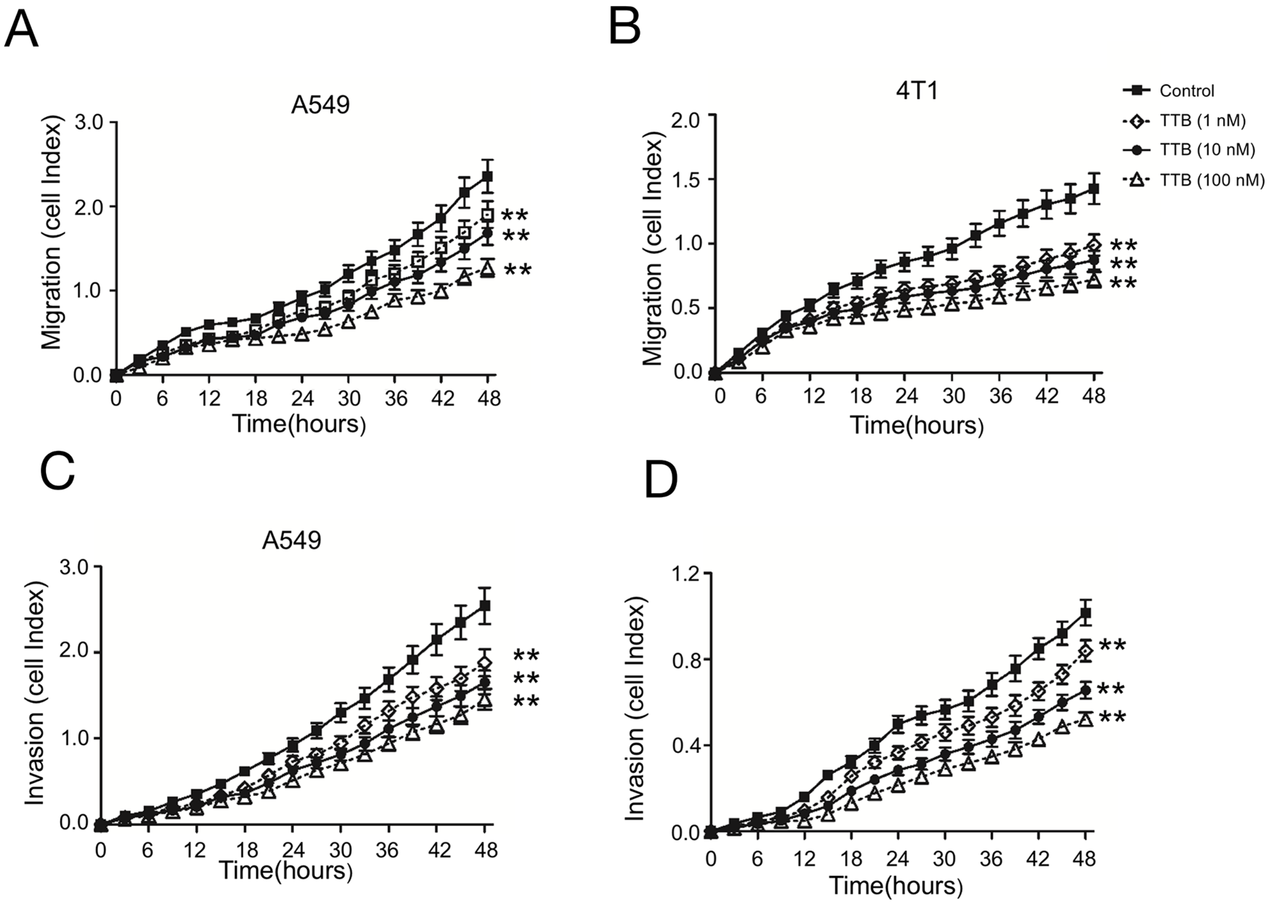

Supplementary Figure 3: Mobility of cancer cells detected using RTCA. A549 and 4T1 cells were seeded in E-plates and treated with TTB for 48 hours. Then cell index (CI) was measured every 30 min. **(A)** A549 and **(B)** 4T1 migration assays, **(C)** A549 and **(D)** 4T1 invasion assays were shown. (Control, solid line with square; TTB 1nM, dot line with diamond; TTB 10nM, solid line with filled circle; TTB 100nM, dot line with triangle, $^{**}P<0.01$ vs. control). Experiments have been repeated at least 3 times. The data are shown as the mean ± SEM.

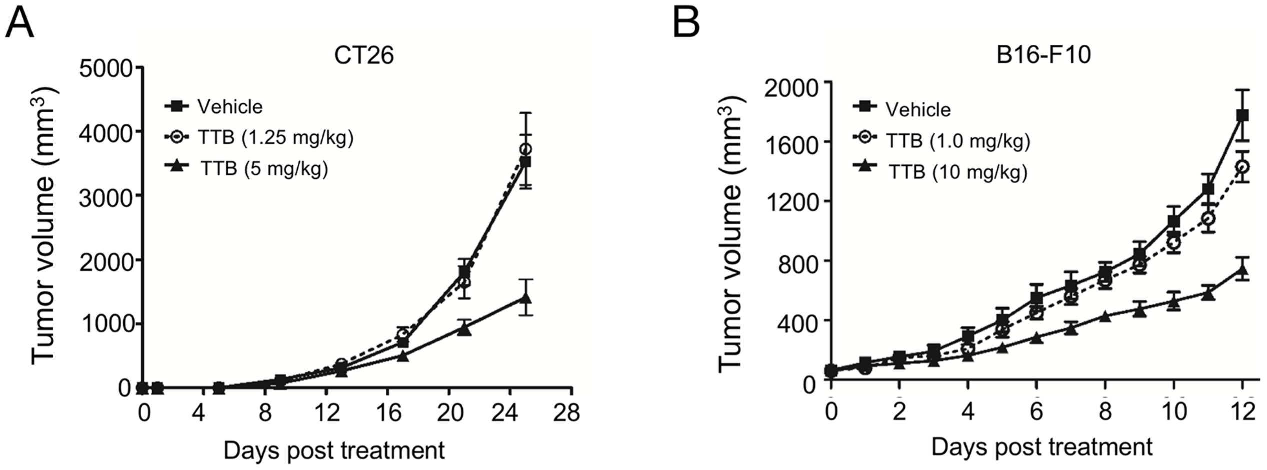

Supplemental Figure 4: Tumor growth inhibition studies were performed as described in the Methods and Materials. **(A)** CT26 cells were s.c. injected to into BALB/c mice (n = 8). Then, mice were intraperitoneally (i.p.) treated with vehicle or TTB every 3 days. The tumor volumes are shown as the mean ± SEM (Vehicle, solid line with square; TTB 1.25mg/kg, dot line with circle; TTB 5mg/kg, solid line with filled circle). **(B)** B16-F10 cells were injected into C57BL/6 mice (n = 8). When tumor volume reached ~100 mm^3, the mice were intraperitoneally (i.p.) treated with vehicle or TTB every 3 days. Growth of the tumors was determined. Experiments have been repeated at least 3 times. The data are shown as the mean ± SEM.

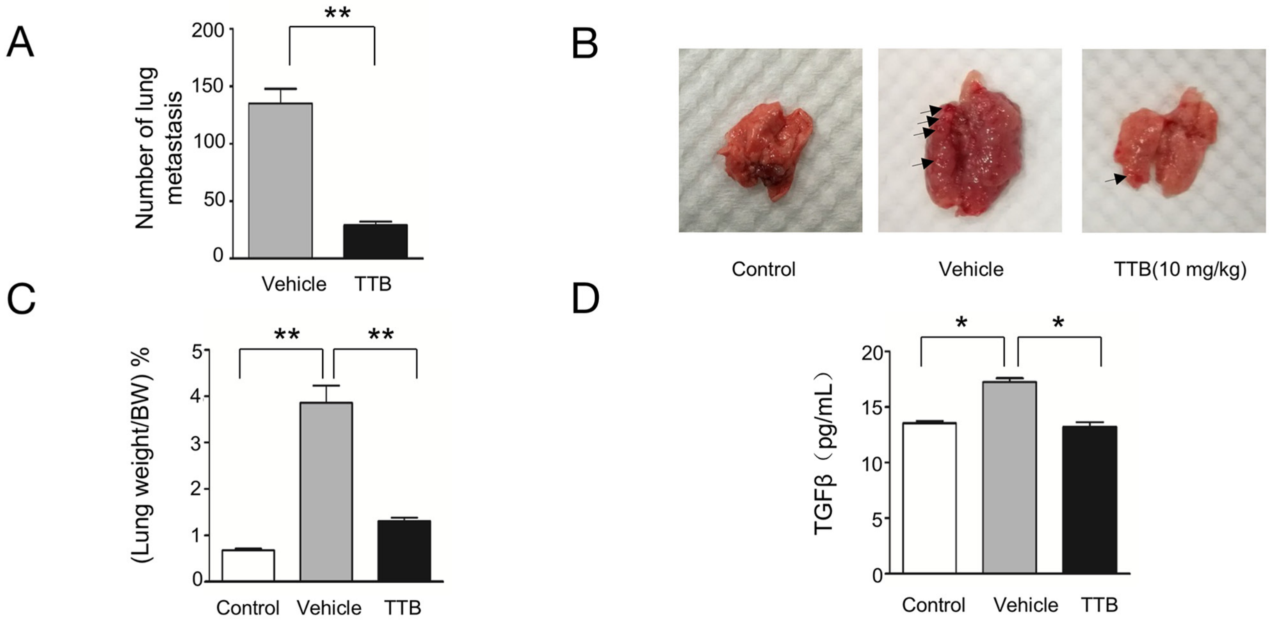

Supplemental Figure 5: Tumor metastasis studies using 4T1 cells injected the left cardiac ventricle of female nude mice. The mice were treated with vehicle or TTB (10mg/kg) by i.p. injection every 3 days. On day 10, the mice were sacrificed. **(A)** The number of lung metastasis, **(B)** the representative lung images, **(C)** the percentage of lung weight/bodyweight, and **(D)** the serum levels of TGF-β, were determined accordingly. The data are shown as the mean ± SEM (*P<0.05, **P<0.01).
